# Modelling the Effects of Weather Conditions on Cereal Grain Contamination with Deoxynivalenol in the Baltic Sea Region

**DOI:** 10.3390/toxins13110737

**Published:** 2021-10-20

**Authors:** Katarzyna Marzec-Schmidt, Thomas Börjesson, Skaidre Suproniene, Małgorzata Jędryczka, Sigita Janavičienė, Tomasz Góral, Ida Karlsson, Yuliia Kochiieru, Piotr Ochodzki, Audronė Mankevičienė, Kristin Piikki

**Affiliations:** 1 Department of Soil and Environment, Swedish University of Agricultural Sciences, 532 23 Skara, Sweden; Kristin.Piikki@slu.se; 2 Agroväst Livsmedel AB, 532 23 Skara, Sweden; thomas.borjesson@agrovast.se; 3 Lithuanian Research Centre for Agriculture and Forestry, LT-58344 Kėdainiai, Lithuania; Skaidre.Suproniene@lammc.lt (S.S.); Sigita.Janaviciene@lammc.lt (S.J.); Yuliia.Kochiieru@lammc.lt (Y.K.); Audrone.Mankeviciene@lammc.lt (A.M.); 4 Department of Pathogen Genetics and Plant Resistance, Institute of Plant Genetics, Polish Academy of Sciences, 60-479 Poznań, Poland; mjed@igr.poznan.pl; 5 Department of Plant Pathology, Plant Breeding and Acclimatization Institute-National Research Institute, Radzików, 05-870 Błonie, Poland; t.goral@ihar.edu.pl (T.G.); p.ochodzki@ihar.edu.pl (P.O.); 6 Department of Crop Production Ecology, Swedish University of Agricultural Sciences, 750 07 Uppsala, Sweden; ida.karlsson@slu.se

**Keywords:** deoxynivalenol (DON) prediction, Fusarium head blight—FHB, machine learning, mycotoxins, phenological development, small grain cereals, Spearman’s rank correlation coefficient

## Abstract

Fusarium head blight (FHB) is one of the most serious diseases of small-grain cereals worldwide, resulting in yield reduction and an accumulation of the mycotoxin deoxynivalenol (DON) in grain. Weather conditions are known to have a significant effect on the ability of fusaria to infect cereals and produce toxins. In the past 10 years, severe outbreaks of FHB, and grain DON contamination exceeding the EU health safety limits, have occurred in countries in the Baltic Sea region. In this study, extensive data from field trials in Sweden, Poland and Lithuania were analysed to identify the most crucial weather variables for the ability of *Fusarium* to produce DON. Models were developed for the prediction of DON contamination levels in harvested grain exceeding 200 µg kg^−1^ for oats, spring barley and spring wheat in Sweden and winter wheat in Poland, and 1250 µg kg^−1^ for spring wheat in Lithuania. These models were able to predict high DON levels with an accuracy of 70–81%. Relative humidity (RH) and precipitation (PREC) were identified as the weather factors with the greatest influence on DON accumulation in grain, with high RH and PREC around flowering and later in grain development and ripening correlated with high DON levels. High temperatures during grain development and senescence reduced the risk of DON accumulation. The performance of the models, based only on weather variables, was relatively accurate. In future studies, it might be of interest to determine whether inclusion of variables such as pre-crop, agronomic factors and crop resistance to FHB could further improve the performance of the models.

## 1. Introduction

Weather conditions significantly affect the life cycle of toxigenic fungi and determine the interaction between host and pathogen, and thus have a significant effect on crop resistance to various toxigenic species and a pathogen’s ability to produce mycotoxins [1,2]. Fusarium head blight (FHB) is a disease of small-grain cereals caused by fungi from the genus *Fusarium*. The main causal agents of FHB in Europe are *Fusarium graminearum* Schwabe, *F. avenaceum* (Fr) Sacc. and *F. culmorum* (W.G.Smith) Sacc. [3,4,5]. FHB results in reductions in yield quantity and quality, but also contamination of grain with mycotoxins, e.g., deoxynivalenol (DON), nivalenol (NIV) and zearalenone (ZEA). These mycotoxins produced by *Fusarium* spp. pose a serious health threat to human and animal health [3]. In the European Union (EU), legal limits on the concentrations of many mycotoxins in food and feed were introduced in 2006 [6]. According to those regulations, the maximum permissible concentration of DON in food for human consumption is 1750 μg kg^−1^ for oats and durum wheat, 1250 μg kg^−1^ for other small-grain cereals, 750 μg kg^−1^ for grain used as feed for piglets and 200 μg kg^−1^ for baby food.

The occurrence of *Fusarium* species and their toxins differs depending on location, climate, weather and crop [7,8,9,10]. Some general trends have been reported, e.g., spring cereals seem to be more susceptible to *Fusarium* contamination than winter cereals. Moreover, it is evident that *F. graminearum* has become more common during the past 10 years [11,12,13,14,15,16,17,18]. *Fusarium graminearum* has a higher optimal temperature for growth than *F. culmorum* [19], so the increasing frequency of its occurrence may be attributable to climate change. In the Baltic Sea region, there is growing awareness of *Fusarium* contamination of cereals and, in particular, the production of DON. Field surveys conducted in Northern Europe suggest that the main producer of DON in cereals is *F. graminearum*, while *F. culmorum* plays a lesser role [8,17,20,21,22,23,24,25]. As *F. graminearum* has become more prevalent, high DON concentrations in spring-sown cereals have also become more frequent and a clear correlation between *F. graminearum* and DON contamination in grain has been observed [15,17,23,26].

Surveys of oats (*Avena sativa* L.) in Sweden have shown that *F*. *poae*, *F*. *langsethiae*, *F*. *avenaceum* and *F*. *graminearum* are the most prevalent species [23,27]. In wheat (*Triticum aestivum* L.), *F*. *graminearum*, *F*. *culmorum, F*. *avenaceum*, and *F*. *poae* are reported to be the most common species, with the dominant species varying depending on the year and region [15,24]. The strong correlation observed between *F*. *graminearum* and DON contamination indicate that *F*. *graminearum* is the dominant DON producer in both wheat and oats in Sweden [15,23]. In Sweden, there has been a strong focus on DON contamination in oats since 2011, when about half of all oats grown in Sweden had a DON content too high to be fit for human consumption [28]. Even though, fewer problems with high DON concentrations in grain have been encountered since then, almost all oats produced are still checked for DON contamination, which generates a high cost to farmers and the grain industry.

In Lithuania, FHB outbreaks in recent years have mostly been associated with *F. graminearum* infections, but species like *F. avenaceum, F. poae*, *F. culmorum*, *F. sporotrichioides*, *F. tricinctum, F. langsethiae, Microdochium nivale* and *M. majus* also contribute [10,29,30,31,32]. Similarly to Sweden, Lithuania has experienced increasing problems with DON contamination during the past 10 years, with DON concentrations in spring wheat grain markedly exceeding the EU permissible limit (1250 μg kg^−1^). For example, measured concentrations were 2150–8845 μg kg^−1^ in 2012 [13], 247–10,644 μg kg^−1^ in 2013 [30] and 1962–18,563 μg kg^−1^ in 2017 [33].

In Poland, *F. culmorum* was long considered the most common species causing FHB in wheat [34,35], but in the past 20 years an increase in *F. graminearum* and decrease in *F. culmorum* have been observed [14,18,25,36,37]. Differences in species frequency between years (and regions) are substantial, but *F. graminearum* has dominated in years with severe outbreaks of FHB in Poland [38].

It is predicted that climate change will increase the risk of mycotoxin contamination in food and feed [39,40]. Predicted climate change scenarios differ between regions, but it is expected that higher temperatures and increased precipitation will create more suitable conditions for fusaria infection of cereals, and associated contamination with mycotoxins, in many regions in Europe. According to models developed by van der Fels-Klerx et al. [41,42], climate change will not only affect the weather conditions but also crops and their development rate during the growing season. It is predicted that flowering and full maturation of wheat in Norway, Sweden, Finland and the Netherlands will be 1–2 weeks earlier than at present. The consensus from all modelling studies is that DON concentrations in grain will increase. Moreover, clear geographical differences in DON concentrations between regions are emerging in many countries, particularly Sweden [24] and to some extent Finland [17]. These differences have been attributed to changing weather conditions [15,17,23,43]. Similar trends have been reported for Lithuania, Latvia, Estonia and Poland [44].

Apart from weather conditions, the incidence and severity of FHB also depend partly on agrotechnical conditions, such as crop rotation and crop management [45,46,47,48,49,50,51], including sowing date and density [52] and harvesting time [10,33]. The number of protective measures available to control the genus *Fusarium* is rather limited [53]. However, the application of fungicides from the demethylation inhibitor group at anthesis has been proven to provide effective control of FHB in wheat and barley [54]. Moreover, numerous studies have also examined the potential for biological control, using, e.g., *Trichoderma* [55,56], yeasts [57] and bacteria [58]. The introduction of resistance genes from highly resistant close or wild relatives of cereals can also be an efficient strategy for integrated control of FHB in cereals [59,60]. Despite extensive research, there is however, no one fully effective method of protection against FHB. Therefore, a reliable prediction model to support decision making on, e.g., fungicide application, is needed as part of the integrated pest management (IPM) toolkit.

In a study in Sweden, Persson et al. [26] used daily weather data for 11 km × 11 km grids to predict whether DON levels in oats would be below the maximum permissible limit of 1750 μg kg^−1^. They calculated 14-day means for five weather variables (air temperature, relative humidity, wind direction, wind speed and cloud cover) and the total amount of precipitation in each 14-day period for the whole cultivation season. The dependent variable was the mean DON content in all oat deliveries to the grain trader Lantmännen from each particular grid. In cross-validated multivariate prediction models for the years 2012–2014, the percentage of correct classifications achieved in that study was around 85% [26]. A somewhat lower percentage of correct classifications (60–70%) was achieved by Xu et al. [61] for a model predicting the DON content in wheat using logistic regression. They modelled data from field trials in four different European countries using different windows (5, 10, 15 and 30 days) of weather data recorded immediately after anthesis and immediately before harvest. They found that a 15-day window was the most suitable interval and that including data from a longer period did not improve the models. They also found that weather data for the periods around anthesis and harvest were valuable input variables, with the vapour pressure deficit (VPD) being one of the most valuable predictors in their study [61]. Attempts to combine data from very different climate conditions in one model might have been the reason for the weaker performance of their model. A similar modelling approach has been employed for oats in Norway [62], where correlations between DON content and weather data in individual phenology windows were tested. Two models were developed in that study, one for the prediction of DON in mid-season, to support farmers in decisions on whether to treat a crop with fungicides, and an end-of-season model to identify grain lots with potential food safety problems. The data windows used varied in length from 4 to 24 days depending on the length of different phenological stages [62]. The most valuable data windows were for tillering, inflorescence emergence, heading/flowering, dough development and ripening. Dry weather at tillering and dough development and warm, moist weather at inflorescence emergence/heading/flowering and ripening were correlated with high DON levels. With the best model developed in that study, around 80% of correct classifications was obtained for samples with DON levels above or below 1000 μg kg^−1^ [62]. In a study in Finland, Kaukoranta et al. [50] used data windows on spatially gridded weather variables to predict *Fusarium* toxins and *Fusarium* species in oats collected from around 800 farmers’ fields between 2003 and 2014. The data windows covered 7-day periods from 42 days before anthesis until harvest, moved one day at a time. The variables used were mean temperature, sum of precipitation, weighted duration of high relative humidity and a variable describing the interaction between temperature and relative humidity. The results showed that high temperatures and dry conditions at about 30 days before anthesis, and high precipitation, high relative humidity and high values for the interaction between temperature and relative humidity just before anthesis, were positively correlated with high DON levels [50]. There were clear similarities between those results and findings in the UK and Norway, indicating that comparisons of data from different countries in the Baltic Sea region is justifiable. However, an earlier attempt to compare data from Norway, Sweden and Finland proved unsuccessful [63], possibly because the dataset was very unbalanced by having high DON values only in one region in Norway, as *F. graminearum* had not yet been established in Sweden and Finland, and very few Swedish samples were included.

To summarise, *Fusarium* toxins accumulate in cereal kernels and may cause a serious threat for humans and animals. Their occurrence differs depending on the location, weather conditions and crop. Although some similarities can be found between countries, there are also region-specific differences. Even though there were attempts to develop models predicting DON contamination in Swedish crops [26,63], no modelling was done for data collected in Poland and Lithuania.

The aim of the present study was to explore similarities and differences between models developed using data from field trials in three neighbouring countries in the Baltic Sea region (Sweden, Poland, Lithuania). These countries all have extensive available data from field trials, to which weather models could be fitted. The overall aim was to determine whether it is possible to create prediction models using data from regions with similar climate conditions and *Fusarium* mycobiota.

Specific objectives of the work were to identify weather factors correlated with a high DON content in cereal grain and the growth stages in which the correlations were strongest; to look for consistent patterns in correlations between weather variables and the prevalence of DON contamination across crop species and countries; and to identify suitable algorithms for predicting the risk of DON contamination.

## 2. Results

### 2.1. Association between DON Contamination Level and Weather Conditions

#### 2.1.1. Sweden

For Sweden, relationships between eight weather variables (daily minimum temperature (T_min_), daily mean temperature (T_mean_), daily maximum temperature (T_max_), precipitation (PREC), relative humidity (RH), vapour pressure deficit (VPD), wind speed (WS) and wind direction (WD)), estimated for 14-day windows, and the DON content in spring cereal (oats, barley, wheat) grains at harvest, were analysed using the Spearman’s rank correlation coefficient.

**Figure 1:**
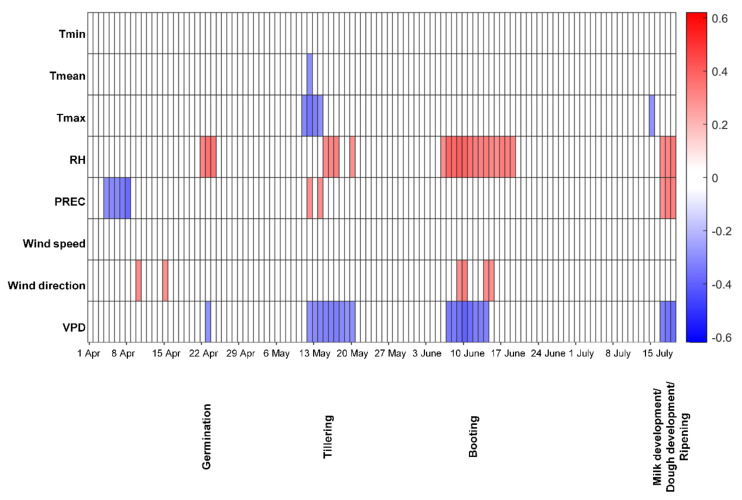
Spearman’s rank correlation coefficient for deoxynivalenol (DON) contamination in Swedish spring oats at harvest and different weather factors estimated for 14-day moving windows during the growing season. Red indicates a positive correlation and blue a negative correlation (both *p* ≤ 0.01) between DON contamination and a particular weather variable, with a darker colour indicating a higher value of the correlation coefficient. Tmin-daily minimum temperature, Tmean-daily mean temperature, Tmax-daily maximum temperature, RH-mean relative humidity, PREC-precipitation, VPD-vapour pressure deficit.

For spring oat grain, the results showed that the DON content was positively correlated with RH around germination (Figure 1). A positive correlation was also observed between DON contamination and RH and precipitation at tillering, but there was a negative correlation between DON and T_max_ and VPD. VPD at booting and later during milk development/dough development/ripening was also negatively correlated with the DON content in spring oat grain. Moreover, high RH at booting and high precipitation during milk development/dough development/ripening, resulted in higher grain contamination with DON.

**Figure 2:**
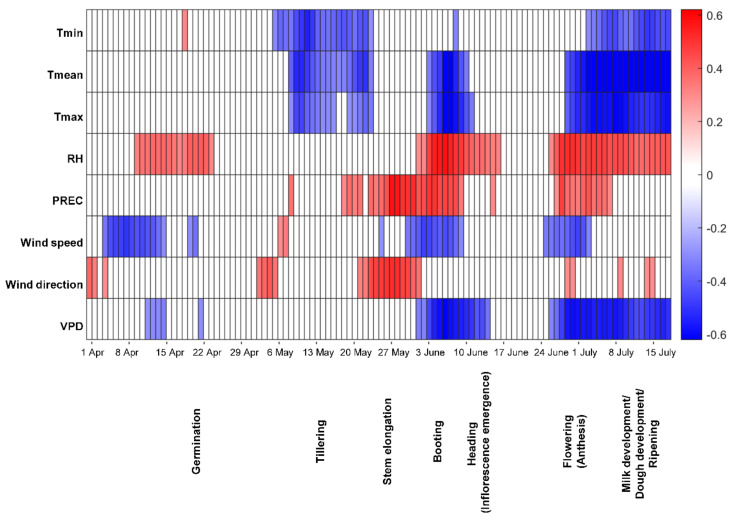
Spearman´s rank correlation coefficient for deoxynivalenol (DON) contamination in Swedish spring barley at harvest and different weather factors estimated for 14-day moving windows during the growing season. Red indicates a positive correlation and blue a negative correlation (both *p* ≤ 0.01) between DON contamination and a particular weather variable, with a darker colour indicating a higher value of the correlation coefficient. Tmin-daily minimum temperature, Tmean-daily mean temperature, Tmax-daily maximum temperature, RH-mean relative humidity, PREC-precipitation, VPD-vapour pressure deficit.

For spring barley grain, the DON contamination level was positively correlated with RH around germination (Figure 2). High values of all three temperature variables at tillering were negatively correlated with DON contamination. High precipitation during the stem elongation stage was associated with a high DON content in grain, as were high RH and precipitation at booting. A negative correlation was found between VPD at heading, flowering and ripening and the DON contamination level, while RH at heading, flowering and ripening was positively correlated with DON contamination, as was precipitation during flowering. T_min_, T_mean_, and T_max_ during flowering and ripening were negatively correlated with the toxin content at harvest.

**Figure 3:**
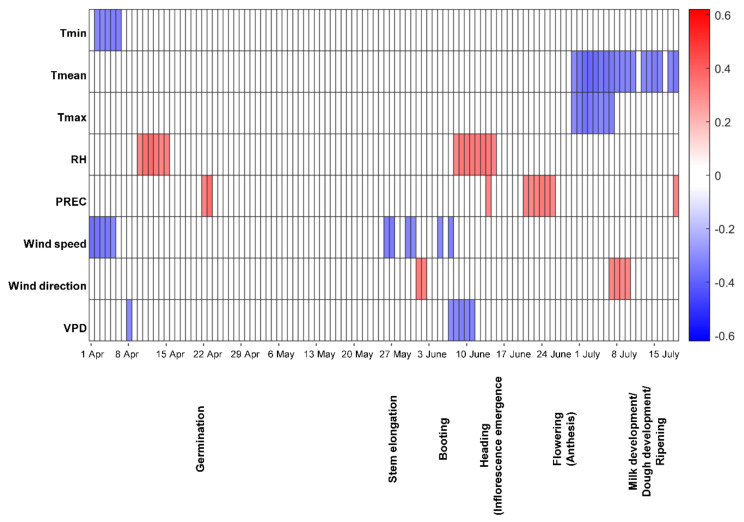
Spearman´s rank correlation coefficient for deoxynivalenol (DON) contamination in Swedish spring wheat at harvest and different weather factors estimated for 14-day moving windows during the growing season. Red indicates a positive correlation and blue a negative correlation (both *p* ≤ 0.01) between DON contamination and a particular weather variable, with a darker colour indicating a higher value of the correlation coefficient. Tmin-daily minimum temperature, Tmean-daily mean temperature, Tmax-daily maximum temperature, RH-mean relative humidity, PREC-precipitation, VPD-vapour pressure deficit.

For spring wheat grain, DON contamination was positively correlated with precipitation around germination (Figure 3). A negative correlation between VPD during booting and DON was observed. RH during the heading stage was positively correlated with DON contamination, as was precipitation during flowering, while the correlation between T_mean_, and T_max_ during flowering and milk development/dough development stages and the toxin content at harvest was negative.

#### 2.1.2. Lithuania

**Figure 4:**
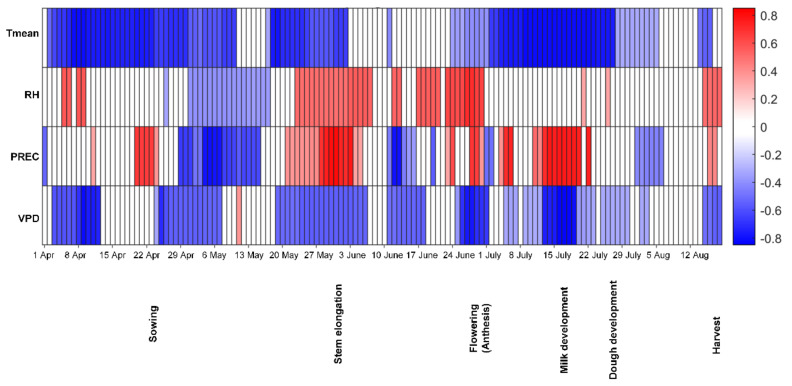
Spearman´s rank correlation coefficient for deoxynivalenol (DON) contamination in Lithuania grown spring wheat at harvest and different weather factors estimated for 14-day moving windows during the growing season. Red indicates a positive correlation and blue a negative correlation (both *p* ≤ 0.01) between DON contamination and a particular weather variable, with a darker colour indicating a higher value of the correlation coefficient. Tmean-daily mean temperature, RH-mean relative humidity, PREC-precipitation, VPD-vapour pressure deficit.

For Lithuania, the relationships between four weather variables (T_mean_, PREC, RH, VPD), estimated for 14-day windows during the growing season, and the DON content in spring wheat grain at harvest were analysed using Spearman’s rank correlation coefficient (Figure 4).

A positive correlation between DON and precipitation and RH during stem elongation, flowering and harvesting was observed, while T_mean_ and VPD during these stages were negatively correlated with the DON level in the harvested grain. There was also a negative correlation between the DON level and T_mean_ and VPD during milk development/dough development, while precipitation during these stages was correlated positively with DON contamination in the harvested grain.

#### 2.1.3. Poland

**Figure 5:**
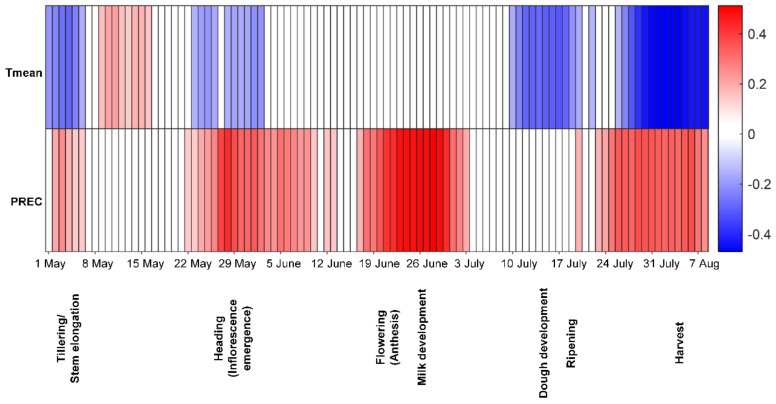
Spearman´s rank correlation coefficient for deoxynivalenol (DON) contamination in Polish winter wheat at harvest and different weather factors estimated for 14-day moving windows during the growing season. Red indicates a positive correlation and blue a negative correlation (both *p* ≤ 0.01) between DON contamination and a particular weather variable, with a darker colour indicating a higher value of the correlation coefficient. Tmean-daily mean temperature, PREC-precipitation.

In Poland, the relationships between two weather variables (T_mean_, PREC), estimated for 14-day windows during the growing season, and the DON content in winter wheat grain at harvest was analysed using the Spearman’s rank correlation coefficient (Figure 5). A positive correlation was found between DON contamination and precipitation during tillering and heading stages, and there was a very strong positive relationship between DON contamination and precipitation during flowering and milk development. Precipitation around harvest was also correlated with a higher DON content in grain (Figure 5). On the other hand, T_mean_ at tillering, stem elongation, dough development, ripening and around harvest was negatively correlated with DON content in the harvested grain.

### 2.2. Development of a Prediction Model to Classify the Risk of DON Contamination

Four models (Decision Tree (DT), Random Forest (RF), Support Vector Machine with Linear Kernel (SVML) and Support Vector Machine with Radial Basis Function Kernel (SVMR)) were used to classify the risk of grain DON contamination >200 µg kg^−1^ (Sweden and Poland) or 1250 µg kg^−1^ (Lithuania). The best models were selected based on their accuracy and sensitivity to predict the DON content. All models were based on the weather variables analysed and on the trial location scale (county in Sweden, district in Lithuania, province in Poland).

#### 2.2.1. Sweden

For oats grown in Sweden, the accuracy of prediction was quite similar for all four models, ranging between 65% (SVMR) and 70% (SVML) (Table 1). However, greater differences were observed in the ability of the models to predict DON levels >200 µg kg^−1^ with accuracy. On taking into consideration all metrics, the models based on the SVML algorithm best predicted the risk of DON contamination at harvest (Table 1).

For SVM models, it may be difficult or even impossible to identify important variables that have the greatest effect on the results. The simpler DT-based model was only slightly less accurate than the SVML model and it allowed variables with the greatest influence on the model to be identified. For the DT model, the most important variables were the region where the oats were grown and the sum of precipitation around seeding.

For Swedish spring barley (*Hordeum vulgare* L.), models based on RF and SVMR performed best, with an accuracy of 77% and 73%, respectively (Table 2). However, significant differences were observed in their sensitivity and specificity, with, e.g., the RF model showing a weaker performance in recognising DON levels >200 µg kg^−1^ and the SVMR model showing a weaker performance in recognising DON levels <200 µg kg^−1^.

**Figure 6:**
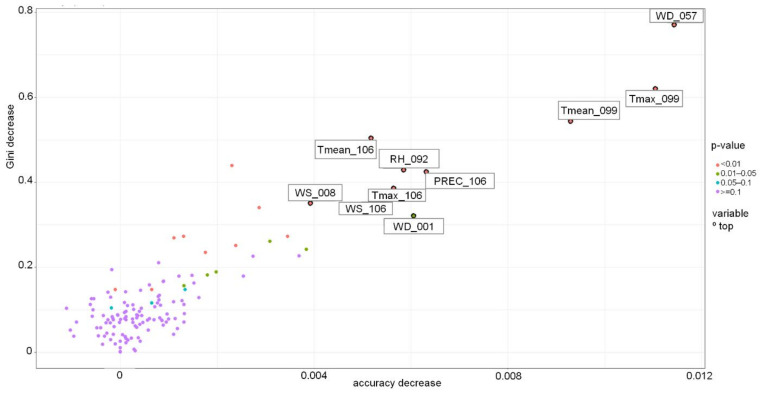
Variable importance in the Random Forest-based model for Sweden grown spring barley. PREC-precipitation, RH-mean relative humidity, Tmax-daily maximum temperature, Tmean-daily mean temperature, WS-mean wind speed, WD-wind direction. PREC_106-PREC 15.07–28.07, RH _092-RH 01.07–14.07, Tmax_099-Tmax 08.07–21.07, Tmax_106-Tmax 15.07–28.07, Tmean_099-Tmean 08.07–21.07, Tmean_106-Tmean 15.07–28.07, WD_001-WD 01.04–14.04, WD_057-WD 27.05–09.06, WS_008-WS 08.04–21.04, WS_106-WS 15.07–28.07.

**Figure 7:**
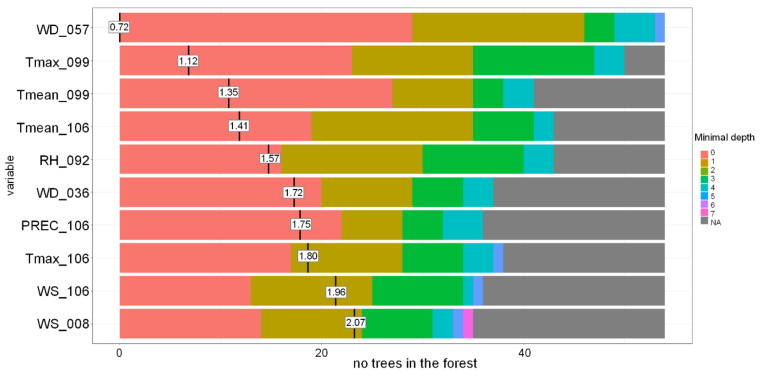
Distribution of the minimal depth of the variable and its mean in the Random Forest-based model for Sweden grown spring barley. PREC-precipitation, RH-mean relative humidity, Tmax-daily maximum temperature, Tmean-daily mean temperature, WS-mean wind speed, WD-wind direction. WD_057-WD 27.05–09.06, Tmax_099-Tmax 08.07–21.07, Tmean_099-Tmean 08.07–21.07, Tmean_106-Tmean 15.07–28.07, RH _092-RH 01.07–14.07, WD_036-WD 06.05–19.05, PREC_106-PREC 15.07–28.07, Tmax_106-Tmax 15.07–28.07, WS_106-WS 15.07–28.07, WS_008-WS 08.04–21.04.

It is possible to identify variables selected as most important by the RF model, e.g., based on their effect on the accuracy and Gini coefficient (Figure 6) or their distribution in the tree (depth) and frequency in the forest (Figure 7). For Swedish spring barley, T_mean_, T_max_, precipitation and RH during late developmental stages (milk development/dough development/ripening) were the most important variables for predicting the grain DON contamination level at harvest.

For spring wheat, the model based on SVMR showed the best accuracy (80%) (Table 3). This model was also able to predict, with an accuracy of 90%, samples with high DON contamination (>200 µg kg^−1^).

**Figure 8:**
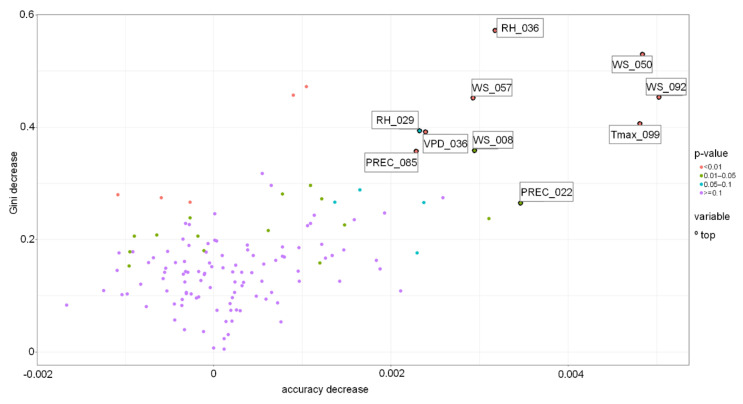
Variable importance in the Random Forest-based model for Sweden grown spring wheat. PREC-precipitation, RH-relative humidity, Tmax-daily maximum temperature, WS-wind speed, WD-wind direction, VPD-vapour pressure deficit. PREC_022-PREC 22.04–05.05, PREC_085-PREC 24.06–07.07, RH _029-RH 29.04–12.05, RH_036-RH 06.05–19.05, Tmax_099-Tmax 08.07–21.07, VPD_036-VPD 06.05–19.05, WS_008-WS 08.04–21.04, WS_050-WS 20.05–02.06, WS_057-WS 27.05–09.06, WS_092-WS 01.07–14.07.

**Figure 9:**
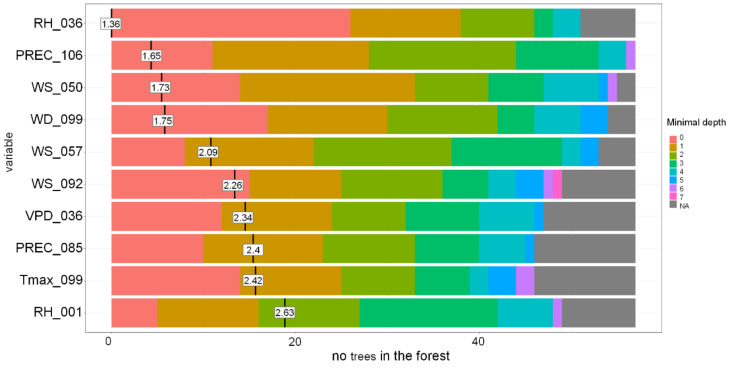
Distribution of the minimal depth of the variable and its mean in the Random Forest-based model for Sweden grown spring wheat. PREC-precipitation, RH-relative humidity, Tmax-daily maximum temperature, WS-wind speed, WD-wind direction, VPD-vapour pressure deficit. RH_036-RH 06.05–19.05, PREC_106-PREC 15.07–28.07, WS_050-WS 20.05–02.06, WD_099-WD 08.07–21.07, WS_057-WS 27.05–09.06, WS_092-WS 01.07–14.07, VPD_036-VPD 06.05–19.05, PREC_085-PREC 24.06–07.07, Tmax_099-Tmax 08.07–21.07, RH _001-RH 01.04–14.04.

The other models were less accurate, with accuracy of around 60–65% (Table 3). For the DT model, the most important variables were the region where the spring wheat was grown and the sum of precipitation around milk development/dough development/ripening. The most important variables for the RF-based model were RH, PREC and VPD during germination and seedling growth, wind speed during tillering and stem elongation, precipitation and flowering, and PREC and T_max_ at the milk development/dough stage (Figure 8 and Figure 9).

#### 2.2.2. Lithuania

For Lithuanian spring wheat, the model based on DT had the highest accuracy (95%) and the highest ability for accurate classification of samples with high and low DON contamination (accuracy 100% and 93%, respectively) (Table 4). The other models performed slightly less well, with accuracy ranging between 84% and 90%, and were significantly weaker in classifying samples with a DON content >1250 µg kg^−1^ (Table 4).

**Figure 10:**
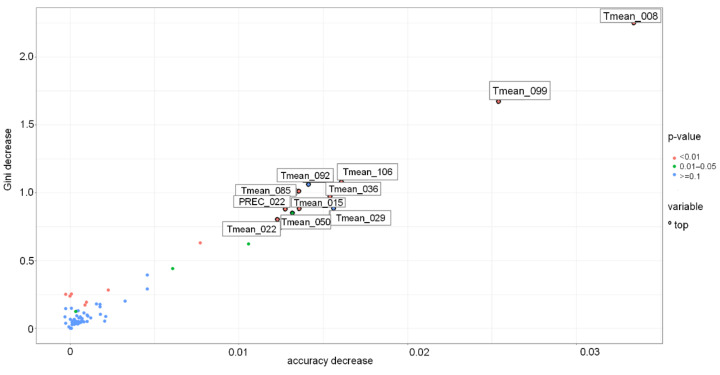
Variable importance in the Random Forest-based model for Lithuania grown spring wheat. PREC-precipitation, Tmean-daily mean temperature. PREC_022-PREC 22.04–05.05, Tmean_008-Tmean 08.04–21.04, Tmean_015-Tmean 15.04–28.04, Tmean_022-Tmean 22.04–05.05, Tmean_029-Tmean 29.04–12.05, Tmean_36-Tmean 06.05–19.05, Tmean_085-Tmean 24.06–07.07, Tmean_092-Tmean 01.07–14.07, Tmean_099-Tmean 08.07–21.07, Tmean_106-Tmean 15.07–28.07.

**Figure 11:**
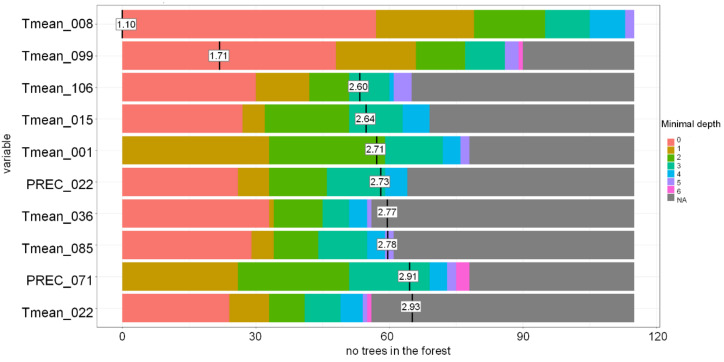
Distribution of the minimal depth of the variable and its mean in the Random Forest-based model for Lithuania grown spring wheat. Tmean-daily mean temperature, PREC-precipitation. Tmean_008-Tmean 08.04–21.04, Tmean_099-Tmean 08.07–21.07, Tmean_106-Tmean 15.07–28.07, Tmean_015-Tmean 15.04–28.04, Tmean_001-Tmean 01.04–14.04, PREC_022-PREC 22.04–05.05, Tmean_036-Tmean 06.05–19.05, Tmean_085-Tmean 24.06–07.07, PREC_071-PREC 10.06–23.06, Tmean_022-Tmean 22.04–05.05.

The DT-based model accurately classified samples based on T_mean_ around sowing and precipitation during stem elongation. According to the RF-based model, the most crucial stages during the growing season were sowing and flowering, when T_mean_ and precipitation were the most important variables, and milk development/dough development/ripening, when T_mean_ strongly affected the DON contamination in the grain at harvest (Figure 10 and Figure 11).

#### 2.2.3. Poland

For winter wheat grown in Poland, the accuracy of prediction was quite similar for all four models, ranging between 69% (SVML) and 75% (DT) (Table 5). However, greater differences were observed in the ability of the models to predict with accuracy DON levels >200 µg kg^−1^. While the DT-based model had the highest accuracy and the highest ability to recognise DON levels <200 µg kg^−1^, it performed worst in identifying samples with high DON contamination levels (Table 5).

For the DT model, the most important variables were precipitation during flowering and milk development/dough development and mean temperature around harvest.

**Figure 12:**
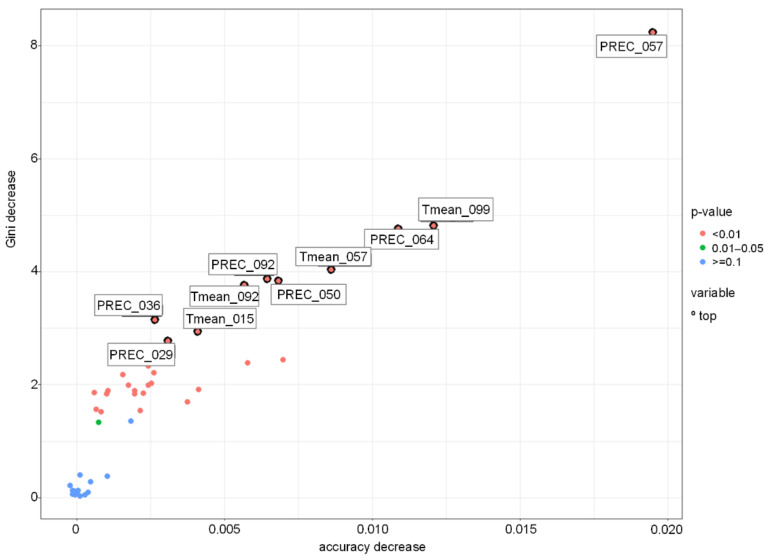
Variable importance in Random Forest-based model for Poland grown winter wheat. PREC-precipitation, Tmean-daily mean temperature. PREC_029-PREC 29.05–11.06, PREC_036-PREC 05.06–18.06, PREC_050-PREC 19.06–02.07, PREC_057-PREC 26.06–09.07, PREC_064-PREC 03.07–16.07, PREC_092-PREC 31.07–13.08, Tmean_015-Tmean 15.05–28.05, Tmean_057-Tmean 26.06–09.07, Tmean092-Tmean 31.07–13.08, Tmean_099-Tmean 08.08–21.08.

**Figure 13:**
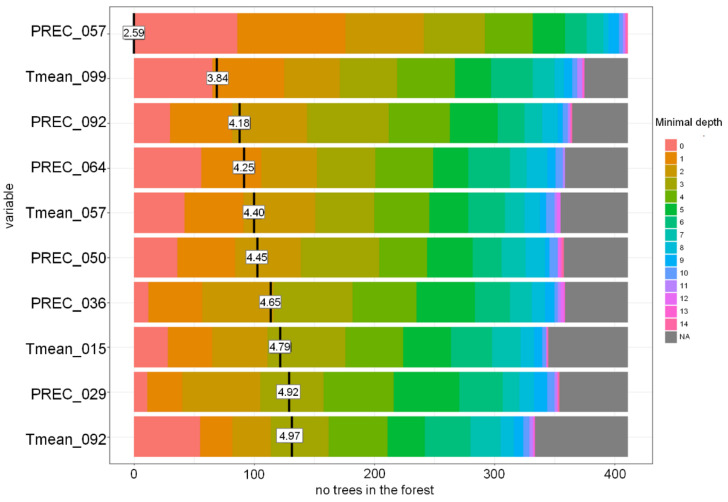
Distribution of the minimal depth of the variable and its mean in the Random Forest-based model for Poland grown winter wheat. PREC-precipitation, Tmean-daily mean temperature. PREC_057-PREC 26.06–09.07, Tmean_099-Tmean 08.08–21.08, PREC_092-PREC 31.07–13.08, PREC_064-PREC 03.07–16.07, Tmean_057-Tmean 26.06–09.07, PREC_050-PREC 19.06–02.07, PREC_036-PREC 05.06–18.06, Tmean_015-Tmean 15.05–28.05, PREC_029-PREC 29.05–11.06, Tmean092-Tmean 31.07–13.08.

The other three models showed rather similar accuracy. The RF model was better at recognising lower DON levels, while the SVM models performed better in recognising DON contamination levels >200 µg kg^−1^ (Table 5). Among the most important variables for the RF-based model were precipitation during heading and flowering, and precipitation and T_mean_ during milk development/dough development/ripening (Figure 12 and Figure 13).

## 3. Discussion

The aim in this study was to develop models for the prediction of DON contamination risk in cereal crops, based on the weather conditions specific for countries in the Baltic Sea region. Field experiments with spring oats, spring barley and spring wheat were conducted during 2010–2014 in 15 counties across Sweden. In Lithuania, field experiments with spring wheat were conducted during 2013–2018 in seven districts. In Poland, field experiments with winter wheat were conducted during 2010–2018 in 16 provinces. The DON content in harvested grain was tested for each field experiment and weather data were taken from the nearest weather station. Models, mainly based on machine learning methods, were developed and tested to predict the risk of high DON accumulation based on the weather variables and geographical location (county in Sweden, district in Lithuania, province in Poland). The four models tested, based on Decision Tree, Random Forest, and Support Vector Machine with Linear or Radial Basis Function Kernel algorithms, showed good overall performance across all data used in this study. Moreover, they revealed the most important weather variables during certain plant developmental stages, allowing the most crucial periods for correlation between DON accumulation in grain and weather conditions to be identified for different crops and locations. Such knowledge is important for assessing the risk of DON contamination, decision making on fungicide application and identifying (at purchase) grain lots with potential food safety problems.

According to Hjelkrem et al. [62], the risk of high DON accumulation in oats in Norway is increased by rainy and humid weather during booting, inflorescence emergence and heading/flowering. Whereas moist and wet conditions during germination/seedling growth and tillering, and cool, moist and wet weather during flowering and later in the season, are negatively correlated with DON contamination. The latter was confirmed in the present study. For oats in Sweden, it was observed that precipitation and RH had the greatest effect on DON accumulation in grain. According to our studies, high values of either variable at germination, seedling growth/tillering, stem elongation/booting/heading and milk development/dough development/ripening is correlated with increased DON contamination. No correlation was seen between rainy and humid weather at flowering and DON contamination in oat grain, possibly because the flowering period in oats is longer and more difficult to identify than in wheat [26,64]. Rainy weather during the milk and dough development and ripening stages can increase the wetness of host tissue, favouring mycelial growth [26], explaining why high precipitation and RH at these stages can lead to increased DON contamination. In contrast, high VPD at stem elongation/booting and high T_max_ around seedling growth/tillering and dough development/ripening reduced the risk of DON accumulation in oat grain.

For spring wheat in Sweden, precipitation during germination/seedling growth, heading/flowering and milk development/dough development/ripening was the most important variable positively correlated with a risk of high DON contamination. The DON concentration in wheat depends on moisture factors during flowering [65,66], with heavy rain and high RH in the days preceding flowering (heading) and following flowering (milk development) resulting in increased mycotoxin contamination of grain [67,68,69,70]. A study by Birr et al. [65] found a highly positive correlation between the DON concentration and precipitation and RH during a period of ±3 days around flowering of highly susceptible cultivars of winter wheat in Germany. For the heading stage (10 to 4 days before flowering) the correlations were weaker, while there were no correlations for the milk development stage (4–18 days post-anthesis). For more tolerant cultivars, as for susceptible cultivars, the highest positive correlations were found between DON content and precipitation and RH during the three days preceding and following flowering [65]. The other variable identified as important in the present study was T_max_ during milk development/dough development/ripening, with a higher T_max_ during these growth stages resulting in a reduced risk of a high DON content.

For spring barley in Sweden, the variables identified as important for a high risk of DON accumulation were high RH at flowering/milk development/dough development, while high T_max_ and T_mean_ around milk development/dough development/ripening decreased the risk. Some similarities between spring wheat and spring barley were observed, with both crops being susceptible to the effect of precipitation during flowering and grain filling stages, and to the effect of temperature during late stages of development.

For spring wheat in Lithuania, high precipitation at tillering/stem elongation was associated with a decreased DON level, while rainy weather during heading, flowering and milk development/dough development/ripening was correlated with an increased risk of high DON contamination. A significant effect of precipitation at flowering on the DON level has been demonstrated in many studies [7,45,65,71]. According to Kochiieru et al. [33], the amount of precipitation around flowering, and at 20–30 days before and 20 days after, is the most important factor for DON contamination of spring wheat grain in Lithuania. Rainy weather during the 2017 harvesting period in Lithuania also resulted in high DON contamination of spring wheat grain, to levels that were several-fold higher than the maximum permissible value set by EU regulations [33]. A high T_mean_ around sowing, flowering and milk development/dough development/ripening was identified as a factor reducing DON contamination in the present study. This is partially consistent with findings by Klem et al. [72] of a negative correlation between DON accumulation in wheat and a high temperature during the five days following flowering. High temperature and low precipitation may lead to reduced moisture availability, resulting in a lower ability of the fungus to sporulate and infect cereal crops. High temperature may also lead to faster development and reduce the length of the flowering stage [62], allowing the crop to ‘escape’ the threat of infection.

For the only winter crop examined in this study, winter wheat in Poland, the most important weather factor was precipitation. High levels of precipitation at flowering, dough development/ripening and around harvest resulted in an increased DON content, which was in line with findings by Birr et al. [65] regarding the effect of weather variables on the DON content in winter wheat in Germany. For winter wheat in Poland, high T_mean_ at heading and the end of development (ripening and harvest) reduced the risk of DON accumulation.

Analysis of the results for all crops in all three Baltic countries identified RH as the factor with a strong influence on DON accumulation in grain. A high RH level during germination, seedling growth, tillering, stem elongation, booting, heading, flowering (spring barley in Sweden, spring wheat in Lithuania), milk development, dough development and ripening (all except spring wheat in Sweden) increased the risk of high DON contamination. Another weather factor of great importance was precipitation, with high precipitation at flowering (all except oats in Sweden), milk development, dough development and around ripening increasing the risk of DON contamination. A high T_max_ during milk development, dough development and ripening also decreased the risk of DON contamination in all three crops in Sweden and in all the spring crops tested in this study. Furthermore, VPD during tillering, stem elongation, heading, booting (all spring crops), flowering, milk development (spring barley in Sweden, spring wheat in Lithuania), dough development, and ripening (all spring crops except wheat in Sweden) was found to be negatively correlated with DON content.

Among the models tested, those based on SVM with either Linear or Radial Basis Function Kernel (SVML, SVMK) performed best overall in predicting the risk of DON contamination based on weather factors and geographical location. Depending on the crop, the accuracy was between 70% and 81%. The DT-based model performed better only for spring wheat in Lithuania. Similar accuracy ranges were obtained by Hjelkrem et al. [73] on applying classification and regression tree (CART) and K-nearest neighbour (KNN) algorithms to predict the risk of leaf blotch disease in Norwegian spring wheat. It is worth emphasising that all the models tested in the present study tended to overestimate the risk of a high level of DON accumulation (´Sensitivity´ in Table 1, Table 2, Table 3, Table 4 and Table 5). From a practical point of view, it is better to base fungicide application on a model that overestimates the risk of high disease severity/mycotoxin accumulation, rather than to miss applying it when needed. A high infection level as a result of missed fungicide treatment can quickly discourage farmers from using forecasting tools based on a model that underestimates the risk. Moreover, in a real-life situation, decisions on fungicide application are not based solely on model predictions using weather data, as other factors, such as pre-crop, host resistance level and other agronomic factors, are included in the final decision [73].

In the present study, the models were based on weather variables summarised for calendar-based 14-day moving windows, which were related to typical crop growth stages at the dates in question according to expert knowledge in the three countries. This practical approach was the only solution permitted by the dataset, but models based on weather variables for windows related to observed developmental stages might have worked even better. The accuracy of model predictions might also be improved if more factors were included, e.g., the pre-crop level of crop resistance to FHB, field tillage regime and even the soil type. These factors should be investigated in future studies.

## 4. Materials and Methods

### 4.1. Association between the Level of DON Contamination in Grain and the Weather Condition

#### 4.1.1. Field Data

**Figure 14:**
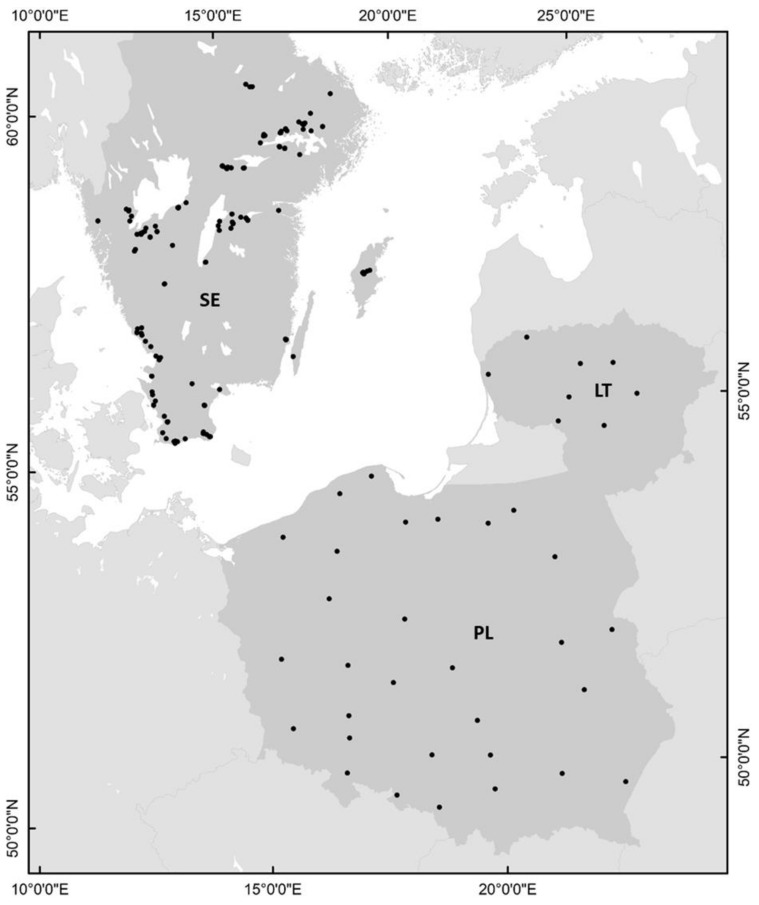
Location of field trials conducted in Sweden (SE), Poland (PL) and Lithuania (LT).

Data on the DON concentration in cereal grain were obtained from controlled field experiments or commercial fields located in Sweden, Lithuania and Poland (Figure 14).

The Swedish data were derived from 203 field trials in 15 Swedish counties between 2010 and 2014, of which 80 trials were on oats, 53 on spring barley and 70 on spring wheat (Table 6). The trials are part of the Swedish Board of Agriculture national monitoring programme for *Fusarium* fungi and their mycotoxins. In Lithuania, 56 spring wheat field experiments and 34 commercial fields in the seven administrative districts included in the monitoring programme conducted by the Lithuanian Research Centre for Agriculture and Forestry during 2013–2018 were selected (Table 6). In Poland, the data used were from 317 winter wheat field trials carried out by the Research Centre for Cultivar Testing (COBORU) at their Variety Testing Stations located in all 16 Polish provinces from 2010 to 2018 (Table 6). Only fields without fungicide application were included in the study.

#### 4.1.2. DON Analysis

Grain samples collected at harvest in Sweden were analysed for DON content using liquid chromatography with tandem mass spectrometry (LC-MS-MS) at Aarhus University (Aarhus, Denmark) according to Nicolaisen et al. [74]. The limit of detection (LOD) of DON was 10 µg kg^−1^. Samples collected in Lithuania and Poland were tested at the Lithuanian Research Centre for Agriculture and Forestry (Akademija, Lithuania) and the Plant Breeding and Acclimatization Institute (IHAR, Radzikow, Poland), using the enzyme-linked immunosorbent assay (ELISA) method with a limit of detection (LOD) below 200 µg kg^−1^. Mycotoxin analysis was performed in duplicate for each sample.

#### 4.1.3. Weather and Environmental Data

In Sweden, weather data, comprising daily minimum air temperature (°C) at a 2 m height above ground level (T_min_), daily mean air temperature (°C) at a 2 m height above ground level (T_mean_), daily maximum air temperature (°C) at a 2 m height above ground level (T_max_), daily precipitation (mm) (PREC), daily relative humidity (%) (RH), daily average wind speed (m s^−1^) and wind direction (deg) measured from 1 April to 31 July, were obtained from nearby weather stations operated by Lantmet or the Swedish Meteorological and Hydrological Institute (SMHI). The vapour pressure deficit (VPD, kPa) was calculated using air temperature and relative humidity values. In Lithuania, weather data, comprising daily mean air temperature (°C) at a 2 m height above ground level (T_mean_), precipitation (mm) (PREC) and daily relative humidity (%) (RH) measured from 1 April to 31 July, were obtained from the nearest weather stations operated by the Lithuanian Hydrometeorological Service. In Poland, weather data, comprising daily mean air temperature (°C) at a 2 m height above ground level (T_mean_) and precipitation (mm) (PREC) measured from 1 May to 31 August, were obtained from weather stations situated at COBORU Variety Testing Stations, where the field experiments were conducted.

During the growing season (1 April–31 July for Sweden and Lithuania, 1 May–31 August for Poland), average daily values in 14-day windows were calculated for each weather variable. Each consecutive 14-day window was moved by one day, giving 110 data windows covering the whole season.

#### 4.1.4. Phenology

In Sweden, data on crop phenology were obtained from the Swedish Board of Agriculture database [75]. These comprised information about the dates of developmental stages through the whole life cycle of oats, spring barley and spring wheat measured in fields in the whole country between 2009 and 2019 (Table 7). In Lithuania, dates of flowering and milk development/dough development for spring wheat were monitored during the field trials in that country (see Field data) (Table 7). In Poland, data about winter wheat phenology stages were obtained from COBORU reports published between 2006 and 2020 [76].

#### 4.1.5. Data Analysis

To identify a possible association between the DON content in grain and the summarised weather variables for the 110 data windows, Spearman´s rank correlation coefficient was calculated. Weather summarisations with a high level of Spearman correlation significance (*p* < 0.01) were recognised as important weather conditions possibly affecting toxin accumulation in cereal grain. A phenological stage was assigned to those significant weather summarisations.

### 4.2. Prediction Model to Classify the Risk of DON Contamination in Grain

#### 4.2.1. Development of a Prediction Model to Classify the Risk of DON Contamination in Grain

Classification models were developed to predict the risk of DON toxin contamination in grain at levels higher than 200 µg kg^−1^ (EU limit for infant and baby food) for Sweden and Poland or higher than 1250 µg kg^−1^ (EU limit for human consumption) for Lithuania based on the weather summarisations for growing seasons and regions. The difference in the DON threshold (high or low) between the countries was due to the differentiated structure of data, with toxin levels below 200 µg kg^−1^ mainly found in trials in Sweden and Poland, but higher values in Lithuania.

After preliminary tests, four machine learning-based algorithms (Random Forest (RF), Decision Tree (DT), Support Vector Machine with Linear Kernel (SVML) and Support Vector Machine with Radial Basis Function Kernel (SVMR)) were chosen for further tests, due to their overall best performance for the datasets used in this study. All four are nonparametric supervised machine learning algorithms. Decision Tree applies a tree-like model starting with a root node on the top of the tree representing the most significant variable, followed by deeper decision nodes, and ends with terminal nodes stating the percentage of certainty for the predicted class. At each branch, the if-then condition is applied to determine the class prediction. Random Forest (Random Decision Forest) was used in this study for classification by constructing multiple decision trees while training and predicting the class based on the number of votes from all trees in the forest. The SVML algorithm creates a line that separates data between two classes. During training, when data are gradually fed into the model, it learns how to separate data belonging to different classes with the widest possible margin. When it is impossible to separate the data linearly, SVMR can be applied instead. In this study, when building the models based on DT and the SVM algorithms, all data were split in such a way that 75% were used for training and 25% for testing. During training, 10-fold cross-validation repeated three times was used as a resampling method. For RF, the dataset was automatically split into 70% of data for training and 30% for testing, and therefore no manual segregation was needed. The default number of trees in the RF was 500 and the number of variables tried at each split was 10.

To reduce the dimensionality of the weather variables, instead of using all 110 data windows covering the whole season (as in Spearman´s rank correlation coefficient), each consecutive 14-day window was moved by 7 days, giving a total of 16 data windows. This reduced the time and computational power needed for training the models, while keeping good data coverage for the growing season.

#### 4.2.2. Model Testing and Comparison

The performance of models based on the DT, RF and SVM algorithms was tested and evaluated using three classification metrics: accuracy, sensitivity (ability to recognise high DON content; >200 µg kg^−1^ for Sweden and Poland, >1250 µg kg^−1^ for Lithuania), and specificity (ability to recognise low DON content; <200 µg kg^−1^ for Sweden and Poland, <1250 µg kg^−1^ for Lithuania). The best classification model for each country was selected based on accuracy.

#### 4.2.3. Identification of the Most Important Variables

When the best classification was obtained using the RF algorithm, it was possible to identify variables most strongly correlated with the risk of high DON accumulation in grain. Variable selection is important in developing and implementing a model, since it helps to understand the biology behind the predictions. The most important variables were selected using (i) variable importance scores based on three feature importance metrics: a decrease in the Gini score (measuring the contribution of each variable to the homogeneity of the nodes and leaves in the random forest); a decrease in the accuracy and *p*-value. Higher values of decrease in the Gini score indicate decreased accuracy, while the lower the *p*-value, the greater the importance of the variable for data classification with the model; and (ii) variable depth, specifying the distribution of the mean minimal depth for each variable and allowing the importance of the variable in the structure and prediction ability of the forest to be assessed. The smaller the mean minimal depth, the more frequently the variable is the root of a tree or close to the root, i.e., it is more significant for the model’s performance.

### 4.3. Software

MATLAB R2020b was used for calculating Spearman´s rank correlation coefficient and creating heatmaps. All models were built and tested using R (version 4.0.3) in the RStudio (version 1.3.1093) environment with the packages randomForest, caret and rpart. The most important variables were identified and graphically presented using randomForestExplainer, tidyverse and tidymodels.

## Figures and Tables

**Table 1:** Performance (accuracy, sensitivity and specificity) of the four models used to predict the risk of a deoxynivalenol (DON) contamination level >200 µg kg^−1^ in Swedish oats, based on the test dataset.

Model	Accuracy (%)	Sensitivity ^1^ (%)	Specificity ^2^ (%)
Decision Tree	68	71	67
Random Forest	66	41	80
Support Vector Machine Linear	70	75	67
Support Vector Machine Radial	65	50	73

^1^ Percentage of predictions correctly classified as DON contamination >200 µg kg^−1^. ^2^ Percentage of predictions correctly classified as DON contamination <200 µg kg^−1^.

**Table 2:** Performance (accuracy, sensitivity and specificity) of the four models used to predict the risk of a deoxynivalenol (DON) contamination level >200 µg kg^−1^ in Swedish spring barley, based on the test dataset.

Model	Accuracy (%)	Sensitivity ^1^ (%)	Specificity ^2^ (%)
Decision Tree	60	80	50
Random Forest	77	63	85
Support Vector Machine Linear	40	60	30
Support Vector Machine Radial	73	80	70

^1^ Percentage of predictions correctly classified as DON contamination >200 µg kg^−1^. ^2^ Percentage of predictions correctly classified as DON contamination <200 µg kg^−1^.

**Table 3:** Performance (accuracy, sensitivity and specificity) of the four models used to predict the risk of a deoxynivalenol (DON) contamination level >200 µg kg^−1^ in Swedish spring wheat, based on the test dataset.

Model	Accuracy (%)	Sensitivity ^1^ (%)	Specificity ^2^ (%)
Decision Tree	65	33	100
Random Forest	60	58	62
Support Vector Machine Linear	60	60	60
Support Vector Machine Radial	80	90	70

^1^ Percentage of predictions correctly classified as DON contamination >200 µg kg^−1^. ^2^ Percentage of predictions correctly classified as DON contamination <200 µg kg^−1^.

**Table 4:** Performance (accuracy, sensitivity and specificity) of the four models used to predict the risk of a deoxynivalenol (DON) contamination level >1250 µg kg^−1^ in Lithuanian spring wheat, based on the test data set.

Model	Accuracy (%)	Sensitivity ^1^ (%)	Specificity ^2^ (%)
Decision Tree	95	100	93
Random Forest	84	74	88
Support Vector Machine Linear	90	83	93
Support Vector Machine Radial	90	83	93

^1^ Percentage of predictions correctly classified as DON contamination >1250 µg kg^−1^. ^2^ Percentage of predictions correctly classified as DON contamination <1250 µg kg^−1^.

**Table 5:** Performance (accuracy, sensitivity and specificity) of the four models used to predict the risk of a deoxynivalenol (DON) contamination level >200 µg kg^−1^ in Polish winter wheat, based on the test data set.

Model	Accuracy (%)	Sensitivity ^1^ (%)	Specificity ^2^ (%)
Decision Tree	75	59	83
Random Forest	71	62	77
Support Vector Machine Linear	69	81	63
Support Vector Machine Radial	70	81	65

^1^ Percentage of predictions correctly classified as DON contamination >200 µg kg^−1^. ^2^ Percentage of predictions correctly classified as DON contamination <200 µg kg^−1^.

**Table 6:** Summary of data on deoxynivalenol (DON) concentration in cereal grain from field trials conducted in Sweden, Poland and Lithuania.

Species	Sweden		Poland		Lithuania	
	All	DON > 200 µg kg^−1^ Grains	All	DON > 200 µg kg^−1^ Grains	All	DON > 1250 µg kg^−1^ Grains
Oats	80	29				
Spring barley	53	19				
Spring wheat	70	36			90	27
Winter wheat			317	108		

**Table 7:** Growth stages with estimated dates and their respective 14-day windows for oats, spring wheat and spring barley grown in Sweden, spring wheat grown in Lithuania, and winter wheat grown in Poland.

Country	Species	Zadoks Growth Scale	Date (dd.mm–dd.mm)	Data Frame
Sweden	oats	Germination GS0	27.04–30.05	DF_022-DF_050
		Seedling growth GS1	05.05–25.05	DF_029-DF_050
		Tillering GS2	11.05–12.06	DF_036-DF_064
		Stem elongation GS3	27.05–29.06	DF_057-DF_078
		Booting GS4	10.06–05.07	DF_071-DF_085
		Heading (Inflorescence emergence) GS5	20.06–13.07	DF_078-DF_092
		Flowering/Polination (Anthesis) GS6	27.06–15.07	DF_085-DF_099
		Milk development GS7	04.07–22.07	DF_092-DF_099
		Dough development GS8	08.07–23.07	DF_092-DF_106
		Ripening GS9	12.07–27.07	DF_092-DF_106
	spring wheat, spring barley	Germination GS0	20.04–17.05	DF_015-DF_036
		Seedling growth GS1	27.04–10.06	DF_022-DF_057
		Tillering GS2	10.05–18.06	DF_036-DF_071
		Stem elongation GS3	21.05–01.07	DF_043-DF_085
		Booting GS4	04.06–10.07	DF_057-DF_092
		Heading (Inflorescence emergence) GS5	11.06–17.07	DF_064-DF_099
		Flowering/Polination (Anthesis) GS6	11.06–23.07	DF_078-DF_106
		Milk development GS7	18.06–22.07	DF_078-DF_106
		Dough development GS8	29.06–27.07	DF_085-DF_106
		Ripening GS9	02.07–27.07	DF_092-DF_106
Lithuania	spring wheat	Flowering, anthesis: Full flowering, 50% of anthers mature GS65	10.06–14.07	DF_071-DF_092
		Milk development GS7	01.07–28.07	DF_092-DF_106
		Dough development GS8	08.07–23.07	DF_099-DF_106
Poland	winter wheat	Tillering GS2/Stem elongation GS3	01.05–14.05	DF_001
		Heading GS5/Flowering GS6 (beginning)	15.05–04.06	DF_015-DF_022
		Flowering GS6/Milk development GS7/Dough development GS8	05.06–25.06	DF_036-DF_043
		Dough development GS8/Ripening GS9	19.06–16.07	DF_050-DF_064
		Harvest	31.07–21.08	DF_092-DF_099

## Data Availability

The data presented in this study are available in this article.
